# Supplementary material for: Switching of a large anomalous Hall effect between metamagnetic phases of a non-collinear antiferromagnet
Source: Sci Rep. 2017 Feb 20;7:42982. doi: 10.1038/srep42982 (PMC5317170; doi:10.1038/srep42982)
Supplement: Supplementary Information [file srep42982-s1.pdf]

Switching of a large anomalous Hall effect between metamagnetic phases of a non-collinear antiferromagnet
Supplementary Information

Christoph Sürgers[1],* Thomas Wolf[2], Peter Adelmann[2],
Wolfram Kittler[1], Gerda Fischer[1], and Hilbert v. Löhneysen[1,2]
[1]*Physikalisches Institut, Karlsruhe Institute of Technology, P.O. Box 6980, 76049 Karlsruhe, Germany and*
[2]*Institut für Festkörperphysik, Karlsruhe Institute of Technology, P.O. Box 3640, 76021 Karlsruhe, Germany*

S1. STRUCTURAL PROPERTIES

In the paramagnetic state, the hexagonal unit cell of Mn_5Si_3 (space group $P6_3/mcm$) with lattice constants $a_h = 0.6910$ nm and $c_h = 0.4814$ nm contains Mn_1 and Mn_2 atoms on two inequivalent lattice sites. The long-range antiferromagnetic order occurring below the Néel temperature T_{N2} is accompanied by a distortion form the hexagonal to an orthorhombic structure with lattice constants $a = 0.6898$ nm, $b = 1.1891$ nm, and $c = 0.4793$ nm [1–4]. $T_{N2} = 100$ K and $T_{N1} = 62$ K have been also determined from susceptibility measurements, and indications of a third anomaly with hysteresis between 30 and 60 K have been found [4].

In the AF2 phase between T_{N2} and T_{N1}, the Mn_1 and one third of the Mn_2 atoms do not exhibit an ordered magnetic moment. The remaining Mn_2 atoms have magnetic moments $\mu \approx 1.5\,\mu_B$ oriented parallel and antiparallel to the crystallographic b axis of the orthorhombic cell in a collinear fashion. In the antiferromagnetic AF1 phase below T_{N1}, the magnetic structure has monoclinic symmetry. In this phase, the atomic positions can still be described with orthorhombic symmetry ($Ccmm$ space group) without inversion symmetry [1, 3, 4]. Here, the Mn_1 atoms acquire an ordered moment presumably due to the expansion of the lattice along the crystallographic c axis and the accompanied increase of the Mn_1-Mn_1 distance [1–4]. The Mn moments point into different directions forming a highly non-collinear antiferromagnetic structure. One third of the Mn_2 atoms still do not exhibit an ordered magnetic moment below T_{N1}.

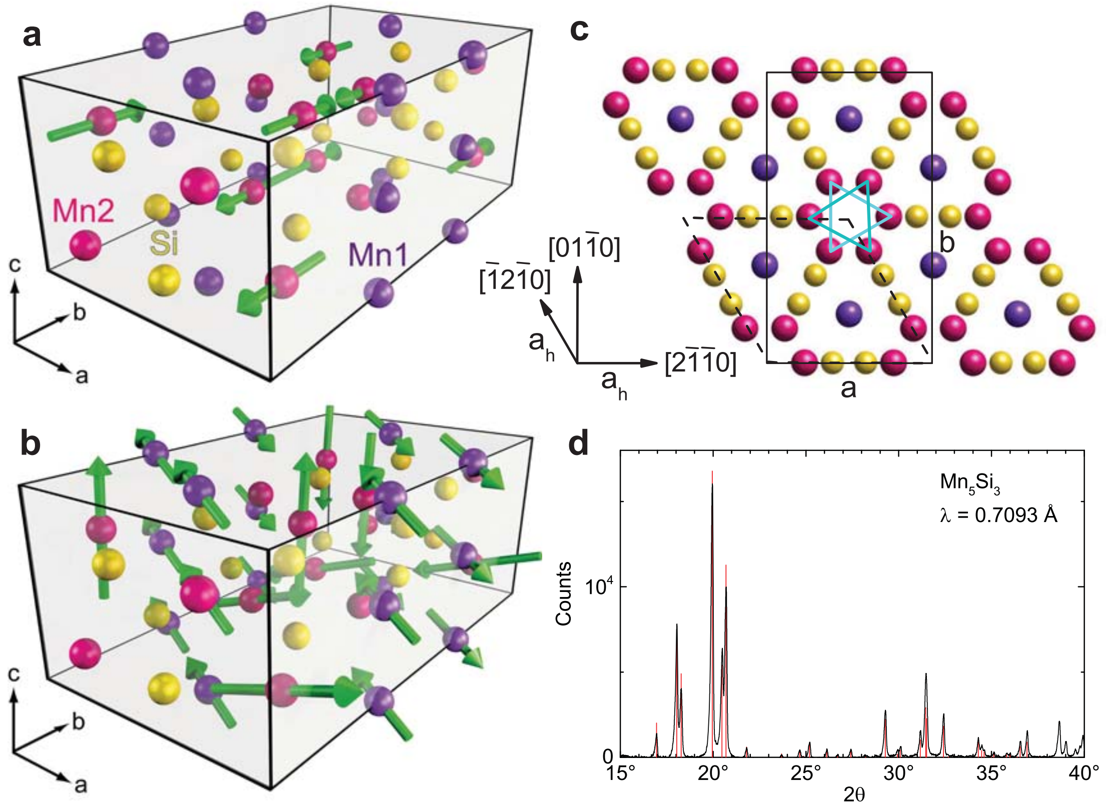

Fig. S1: **a,** Collinear antiferromagnetic structure of Mn_5Si_3 for $T_{N1} < T < T_{N2}$ [2, 4]. **b,** Non-collinear antiferromagnetic structure of Mn_5Si_3 for $T < T_{N1}$ [1]. **c,** View along the crystallographic c axis. Dashed and solid lines indicate the hexagonal and orthorhombic unit cell, respectively, of Mn_5Si_3 above $T_{N2} = 100$ K. The orthorhombic a and b axes correspond to the [2$\bar{1}\bar{1}$0] and [01$\bar{1}$0] directions of the hexagonal structure, respectively, where a_h indicate the axes in the basal plane. The two triangular arrangements of Mn_2 at different heights along c (cyan lines) represent a geometrically frustrated system [4]. **d,** X-ray diffraction pattern of a Mn_5Si_3 powder sample obtained from the same ingot as the measured crystals. Red lines indicate the reflections according to the JCPDS database, entry # 46-1285.

S2. MEASUREMENT OF HALL RESISTIVITY AND MAGNETORESISTIVITY

The Hall resisistivity was measured with a four-point or five-point probe method, depending on the sample size. In the latter, a longitudinal voltage was added on the positive Hall-voltage side to compensate for the offset due to the longitudinal resistance arising from misaligned Hall-voltage probes. However, because of the strong variation of the resistivity at the high-field transition (≈ 10 %), the raw data of the Hall resistivity shows strong varia-

tions for fields close to the transition. Therefore, the Hall effect and longitudinal resistivity were symmetrized by the following procedure and by taking into account the direction of the measurement loop.

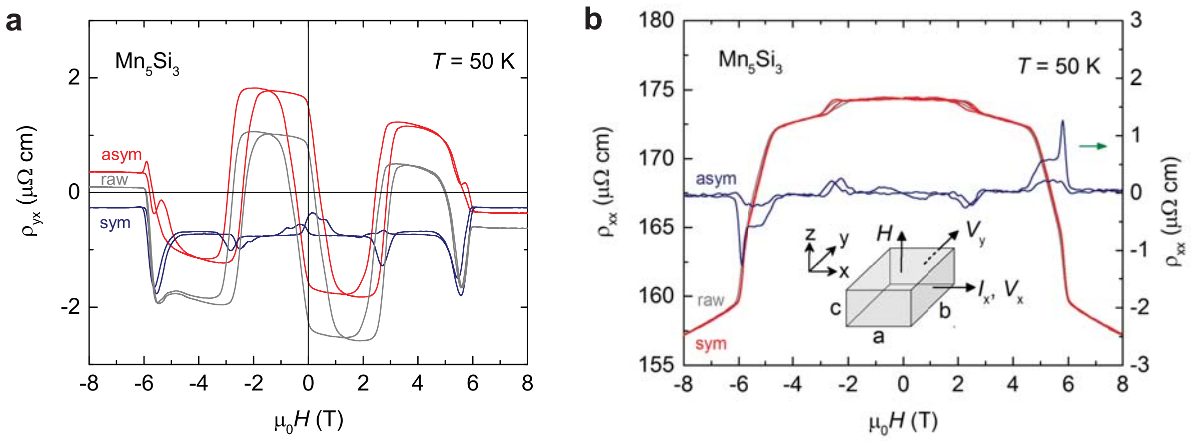

Fig. S2: **a,** Hall resistivity $\rho_{\rm yx}$ for H along the crystallographic c axis at $T = 50$ K. Grey: raw data, red: asymmetric part, blue: symmetric part. **b,** Magnetoresistivity $\rho_{\rm xx}(H)$. Grey: raw data, red: symmetric part, blue: asymmetric part (right scale).

We assume that the resistivity is an even function of magnetic field, $\rho_{\rm xx}(+H) = \rho_{\rm xx}(-H)$, whereas the Hall effect is an odd function of magnetic field, $\rho_{\rm yx}(+H) = -\rho_{\rm yx}(-H)$. The asymmetric average of the measured Hall resistivity $\rho^{\rm m}_{\rm yx}$ gives $\rho_{\rm yx}(H) \approx [\rho^{\rm m}_{\rm yx}(+H) - \rho^{\rm m}_{\rm yx}(-H)]/2$ and the symmetric average of the measured magnetoresistivity $\rho^{\rm m}_{\rm xx}$ gives $\rho_{\rm xx}(H) \approx [\rho^{\rm m}_{\rm xx}(+H) + \rho^{\rm m}_{\rm xx}(-H)]/2$. Fig. S2 shows that this procedure does not change the qualitative behaviour but removes some unusual dips and spikes of $\rho^{\rm m}_{\rm yx}(H)$ close to the metamagnetic transition at $\approx$ 5.5 T where $\rho_{\rm xx}$ changes considerably.

S3. HALL RESISTIVITY AND MAGNETIZATION FOR MAGNETIC FIELDS ALONG THE ORTHORHOMBIC b AND a AXES

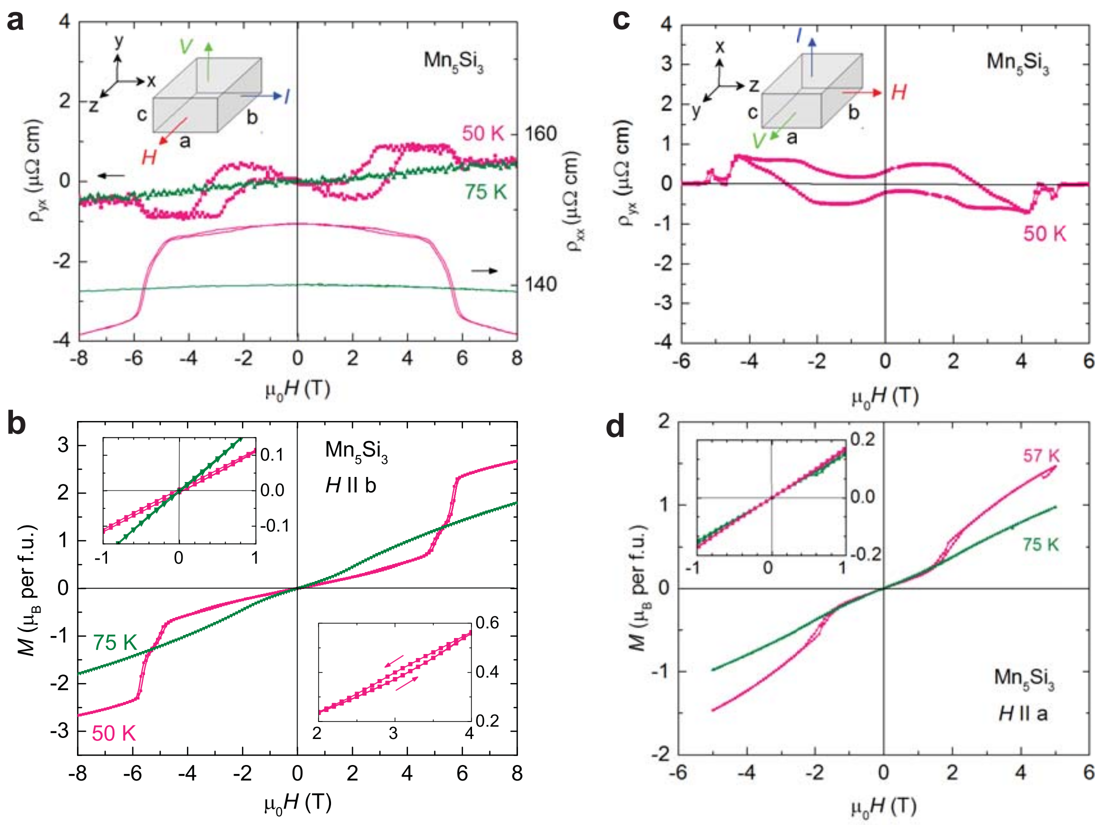

Fig. S3: **a,** Hall resistivity and magnetoresistivity of Mn_5Si_3 for H along the orthorhombic b axis at 50 and 75 K. **b,** Magnetization for H along the orthorhombic b axis at 50 and 75 K. Inset show data for a restricted range of magnetic field. **c,** Hall resistivity for H along the orthorhombic a axis. **d,** Magnetization for H along the orthorhombic a axis at 57 and 75 K. Inset shows $M(H)$ at magnetic fields close to zero.

S4. TEMPERATURE DEPENDENCE OF RESISTIVITY

The longitudinal resistivity ρ_{xx} in perpendicular magnetic field shows a characteristic behaviour already reported for this material [5]. In zero field, ρ_{xx} exhibits two broad humps below T_{N2} and T_{N1} due to the formation of magnetic superzones in the antiferromagnetic state. Similar behaviour is observed when the field is oriented along the crystallographic b axis [6]. At temperatures below $\approx$ 15 K, a behaviour $\rho_{\mathrm{xx}} \propto T^2$ as observed in polycrystalline

films [7] due to scattering of electrons by spin waves with strong spin-wave stiffness. For magnetic fields oriented along the crystallographic a and b axes these two humps disappear and only small kinks are left at T_{N2} and T_{N1}, Fig. S4b,c. The discontinuities at T_{N2} remain independent of the applied magnetic field while T_{N1} decreases toward lower temperatures with increasing field.

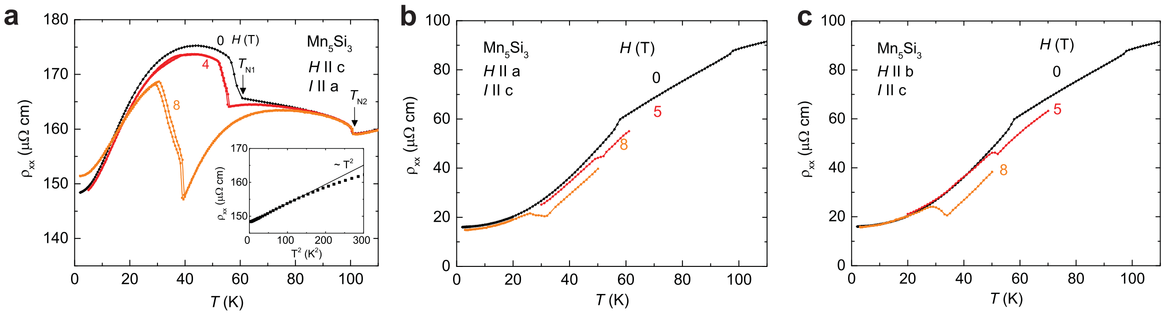

Fig. S4: a, Resistivity $\rho_{\rm xx}$ for H along the crystallographic c axis and I along the a axis. Inset shows a plot $\rho_{\rm xx}$ vs. T^2 for $H = 0$, solid line indicates a T^2 behaviour. **b,** $\rho_{xx}(H)$ for H along the crystallographic a axis and I along the c axis. **c** $\rho_{xx}(H)$ for H along the crystallographic b axis and I along the c axis.

---

* `christoph.suergers@kit.edu`

[1] Brown, P. J., Forsyth, J. B., Nunez, V. & Tasset, F. The low-temperature antiferromagnetic structure of Mn_5Si_3 revised in the light of neutron polarimetry. *J. Phys.: Condens. Matter* **4**, 10025 (1992). URL `http://iopscience.iop.org/0953-8984/4/49/029`.

[2] Brown, P. J. & Forsyth, J. B. Antiferromagnetism in Mn_5Si_3: the magnetic structure of the AF2 phase at 70 K. *J. Phys.: Condens. Matter* **7**, 7619 (1995). URL `http://iopscience.iop.org/0953-8984/7/39/004`.

[3] Silva, M. R., Brown, P. J. & Forsyth, J. B. Magnetic moments and magnetic site susceptibilities in Mn_5Si_3. *J. Phys.: Condens. Matter* **14**, 8707 (2002). URL `http://iopscience.iop.org/0953-8984/14/37/307`.

[4] Gottschilch, M. *et al.* Study of the antiferromagnetism of Mn_5Si_3: an inverse magnetocaloric effect material. *J. Mater. Chem.* **22**, 15275–15284 (2012). URL `http://pubs.rsc.org/en/content/articlelanding/2012/jm/c2jm00154c`.

[5] Vinokurova, L., Ivanov, V., Kulatov, E. & Vlasov, A. Magnetic phase transitions and electronic structure of the manganese silicides. *Journal of Magnetism and Magnetic Materials* **90 & 91**, 121–125 (1990). URL http://www.sciencedirect.com/science/article/pii/S030488531080040X.

[6] Sürgers, C., Kittler, W., Wolf, T. & Löhneysen, H. v. Anomalous Hall effect in the noncollinear antiferromagnet Mn_5Si_3. *AIP Advances* **6**, 055604 (2016). URL http://scitation.aip.org/content/aip/journal/adva/6/5/10.1063/1.4943759.

[7] Gopalakrishnan, B. *et al.* Electronic transport in magnetically ordered $Mn_5Si_3C_x$. *Phys. Rev. B* **77**, 104414 (2008).
